# Virtual Education in Urogynecology: Enhancing Understanding and Management of Pelvic Fistulas

**DOI:** 10.15766/mep_2374-8265.11407

**Published:** 2024-06-04

**Authors:** Margot Le Neveu, Jacqueline Y. Kikuchi, Lindsay R. Ledebur, Jaime B. Long, Erica Qiao, Chi Chiung Grace Chen

**Affiliations:** 1 Fourth-Year Resident, Department of Gynecology and Obstetrics, Johns Hopkins University School of Medicine; 2 Fellow, Department of Gynecology and Obstetrics, Johns Hopkins University School of Medicine; 3 Instructional Designer, Office of Online Education, Johns Hopkins University School of Medicine; 4 Assistant Professor, Department of Obstetrics and Gynecology, Penn State College of Medicine; 5 Fourth-Year Medical Student, University of Washington School of Medicine; 6 Associate Professor, Department of Gynecology and Obstetrics, Johns Hopkins University School of Medicine

**Keywords:** Fistulas, Pelvic Floor Dysfunction, Case-Based Learning, Computer-Based Simulation, OB/GYN, Online/Distance Learning, Self-Regulated Learning

## Abstract

**Introduction:**

Pelvic fistulas affect a significant number of patients globally, with a relatively low prevalence in the United States. Virtual education offers an effective, scalable solution to bridge this educational gap and lead to a deeper understanding of more common conditions, such as urinary and fecal incontinence.

**Methods:**

We developed two virtual cases on rectovaginal and vesicovaginal/ureterovaginal fistulas to enhance medical students’ exposure, knowledge, and confidence regarding assessment of pelvic fistulas. The cases could be completed in approximately 30 minutes, asynchronously, and at students’ own pace. The cases were integrated into an OB/GYN clerkship. We conducted a survey among students receiving the cases to gather feedback on usability, acceptability, and educational value, which guided subsequent improvements.

**Results:**

Forty medical students, ranging from first to third year, participated in the urogynecology elective; 21 (53%) completed the survey. Ninety-one percent agreed or strongly agreed they were satisfied with the cases. All respondents found the format easy to use and appropriate for their level of learning. Most reported the cases improved their confidence in nonsurgical and surgical management options for pelvic fistulas.

**Discussion:**

Offering virtual and interactive patient cases on e-learning platforms represents an innovative approach to increasing clinical exposure to urogynecologic disorders. By providing medical students with the opportunity to interact with pelvic fistulas virtually, these cases can help bridge a gap in clinical education. Future exploration is valuable for examining knowledge deficiencies and developing cost-effective, self-paced, easily accessible educational resources to advance medical training and optimize patient care.

## Educational Objectives

By the end of this activity, learners will be able to:

1. Conduct a thorough patient interview and obtain an appropriate history for rectovaginal and vesicovaginal/ureterovaginal fistulas.
2. Describe the components of a gynecologic and urogynecologic physical examination.
3. Formulate a differential diagnosis for rectovaginal and vesicovaginal/ureterovaginal fistulas.
4. Describe the various surgical and nonsurgical treatment options for rectovaginal and vesicovaginal/ureterovaginal fistulas.
5. Increase their confidence in caring for patients with rectovaginal and vesicovaginal/ureterovaginal fistulas.

## Introduction

The incidence of gynecologic fistulas in developed nations is estimated to be 0.06%.^1^ While fistula due to birth trauma is less common in these countries, fistulas can also result as a sequela of surgery, inflammatory bowel disease, radiation, and malignancy. In the United States, urinary tract fistulas most commonly occur after hysterectomy, with an estimated incidence of 0.1%-0.5%.^2–4^

Due to the low prevalence of these diseases, medical students and OB/GYN residents often have limited exposure to the diagnosis and management of pelvic fistulas, resulting in a clinical education gap.^5^ Virtual education is a cost-effective and scalable option for medical schools and residencies, regardless of resources or intended audience. It can provide personalized learning, can supplement traditional teaching methods by offering interactive, self-paced study, and has been found to increase student engagement.^6^ Enhancing clinical education surrounding pelvic fistulas may also lead to a better understanding of more commonly encountered pelvic floor conditions, including urinary incontinence and accidental bowel leakage.

While there is a growing body of literature examining the effectiveness of virtual medical education tools for various pelvic floor disorders, such as cases previously published by our research team (Kikuchi and colleagues),^7^ limited research has been done on their use specifically for improving medical student awareness of pelvic fistulas. Based on a recent review of PubMed, Google Scholar, and the Association of Professors of Gynecology and Obstetrics website, the cases published by Kikuchi and colleagues are the only publicly available virtual education modules for the management of pelvic floor disorders, and there are no currently available cases that cover the evaluation and treatment of pelvic fistulas, including vesicovaginal fistula, ureterovaginal fistula, and rectovaginal fistula.^7^

We aimed to make two virtual and interactive pelvic fistula cases publicly available and to demonstrate the benefits of virtual medical education as an effective learning tool. The goal of making these resources and educational materials readily available was so medical learners could develop confidence in their ability to identify risk factors for, to diagnose, and to manage the clinical care of pelvic floor disorders and pelvic fistulas, ultimately leading to improved recognition and care for affected patients.

## Methods

We developed virtual and interactive urogynecologic patient cases on the Rise 360 course e-learning platform (Articulate Global) to increase medical student exposure to pelvic fistulas and improve their ability to diagnose and manage these conditions. The patient cases emphasized clinical reasoning, prompting students to conduct a thorough history, outline the components of a urogynecologic physical exam, consider a broad differential diagnosis, and formulate a treatment plan. The cases were each designed to be completed in approximately 30 minutes but could be completed asynchronously and at the students’ own pace.

### Creation of Virtual Patient Cases

The urogynecology physicians from the Johns Hopkins University School of Medicine and the Penn State College of Medicine created two interactive virtual patient cases. These cases were designed to simulate real-world patient encounters and provide participants with an opportunity to interact with and diagnose virtual patients with pelvic fistulas.

The virtual patient cases included the following:

• Mrs. Smith - rectovaginal fistula (Appendix A): Patient presented with persistent brown vaginal discharge. The case prompted students to obtain a history of present illness related to the chief complaint and inquire about pertinent histories (medical, obstetric, gynecologic, surgical, family, social, allergies, and medications). The case also prompted them on components of a focused physical exam and reviewed pertinent findings on rectal, speculum, pelvic, and urogynecologic exam. The case included results of relevant tests (blue-gel rectal test) and the role of other imaging studies (barium enema, pelvic MRI, anal manometry, and endoanal ultrasound). The case reviewed a differential diagnosis and risk factors for developing a rectovaginal fistula. Finally, nonsurgical treatment and options for surgical repair were outlined.
• Mrs. Lopez - vesicovaginal fistula or ureterovaginal fistula (Appendix B): Patient presented with persistent wetness. The case prompted students to obtain a history of present illness related to the chief complaint and inquire about pertinent histories (medical, obstetric, gynecologic, surgical, family, social, allergies, and medications). The case also prompted them on components of a focused physical exam and reviewed pertinent findings on speculum, pelvic, and urogynecologic exam. The results of relevant diagnostic tests (tampon-dye test, postvoid residual, urinalysis, and cystourethroscopy) were also presented. The role of other imaging studies was discussed. The case reviewed a differential diagnosis and risk factors for developing a vesicovaginal fistula or ureterovaginal fistula. Finally, nonsurgical management and options for surgical repair were outlined.

### Implementation of Cases

After conducting a pilot study with students enrolled in a urogynecology elective, we integrated these cases into the medical student OB/GYN clerkship at the Johns Hopkins University School of Medicine and the Penn State College of Medicine at the beginning of the 2020–2021 academic year. Approximately 25% of medical students choose to participate in a urogynecology elective during their OB/GYN clerkship, and all students participating in the 2-week urogynecology rotation were provided with the virtual cases. Students received a guide (Appendix C) explaining expectations and instructions for completing the cases and were encouraged to complete the virtual cases independently during their rotation. Both cases were available to students throughout the entire OB/GYN clerkship. A total of 40 students participated in the urogynecology rotation during the first academic year that the virtual cases were integrated (2020–2021), and a survey was administered to all participants.

### Evaluation

The survey was created with Qualtrics software using 5-point anchored Likert scales and open-ended questions (Appendix D). After completing the virtual patient cases and their OB/GYN clerkship, all participants were asked to anonymously provide feedback on the usability, acceptability, and educational value of the cases. A team member not involved in student evaluation distributed the survey electronically by email at the conclusion of the clerkship. Feedback was used to improve the virtual patient cases and ensure they were effective in achieving the educational goals of the program. The local institutional review board exempted this work.

## Results

All medical students (*N* = 40) who participated in the urogynecology elective within the OB/GYN clerkship between July 2020 and July 2021 received the virtual patient cases. The Qualtrics survey was administered to all participating students, with a response rate of 53% (*n* = 21). Participants included medical students in the first year (*n* = 1), second year (*n* = 3), and third year (*n* = 17). Two surveys remained incomplete at the conclusion of the evaluation.

Students who completed the pelvic fistula virtual cases were the same as those presented in our previous publication; thus, the participant characteristics included in Table 1 match those of the students described by Kikuchi and colleagues.^7^ Students who responded indicated an interest in pursuing a variety of specialties, with few (*n* = 2) reporting an interest in pursuing OB/GYN. Unique feedback specific to evaluation of the fistula cases is presented in Tables 2 and 3.

**Table 1.**
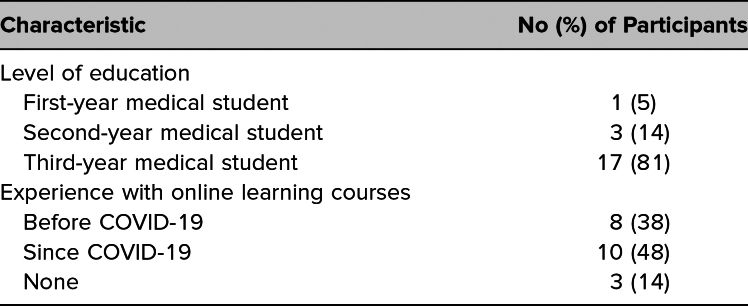
Characteristics of Survey Participants (*N* = 21)

**Table 2.**
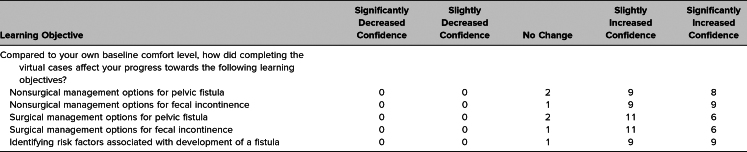
Student Perception of Confidence in the Learning Objectives After Completion of Cases

**Table 3.**
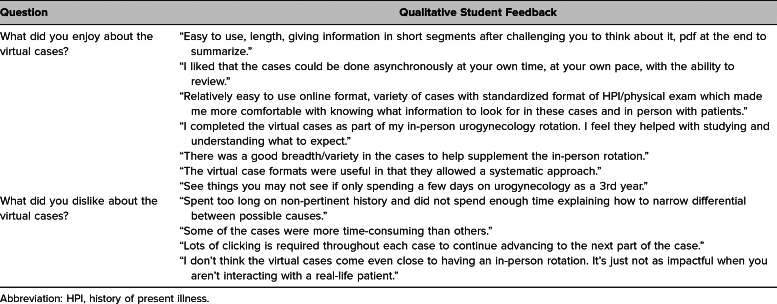
Student Comments on the Cases

All students (100%) who completed the survey felt the virtual cases were useful in advancing their knowledge of urogynecology and pelvic floor disorders, were appropriate for their level of learning, and had a format that was easy to use. Most students felt the format was conducive to accomplishing their learning goals (90%), were overall satisfied with the virtual patient cases (95%), and would recommend these virtual cases to other students (75%).

Most students who completed the survey felt the virtual learning cases increased their confidence in both surgical and nonsurgical management of pelvic fistulas (89%). Student comfort levels towards learning goals related to pelvic fistulas are displayed in Table 2.

Representative qualitative comments are included in Table 3. Students shared similar positive thematic sentiments in their qualitative reflections, citing ease of use (*n* = 7), comprehensiveness (*n* = 3), and accessibility and flexibility (*n* = 3). They also appreciated the cases’ systematic approach (*n* = 3) and breadth of different urogynecologic patient presentations (*n* = 2). Notable areas for improvement included interface and clicking issues (*n* = 5) and limited patient interaction (*n* = 5).

## Discussion

Our virtual patient cases were effective in advancing medical students’ knowledge of and exposure to urogynecology and pelvic floor disorders. Most students felt the virtual learning cases increased their confidence in both surgical and nonsurgical management of pelvic fistulas. All surveyed students found the virtual cases to be useful, appropriate for their level of learning, and presented in a user-friendly format. Overall, students expressed a high level of satisfaction and would recommend these virtual patient cases to their peers. The preidentified educational objectives were met, as most students felt they had improved capabilities to obtain an appropriate patient history, describe components of a urogynecologic physical exam, formulate a relevant differential diagnosis, and describe various treatment options for vesicovaginal/ureterovaginal fistulas. Making these cases widely available may help to address the gap in understanding these uncommonly encountered conditions.

Despite the devastating impact of pelvic fistulas on a woman's quality of life, there is a recognized lack of exposure to their diagnosis and management across all levels of medical training. Even fellows who have successfully completed a female pelvic medicine and reconstructive surgery fellowship report the lowest level of readiness to independently perform vesicovaginal and rectovaginal surgical repairs.^8^ The paucity of pelvic fistula cases highlights a need to address this gap in exposure beginning at the most fundamental level with medical student education. Virtual patient cases provide a valuable opportunity to encounter and manage rare or complex patient scenarios that medical students and other learners encounter less frequently during their medical training. By engaging with these virtual cases, learners can practice and develop their competence and confidence in dealing with such scenarios before encountering patients with rare conditions. Virtual practice can contribute to their preparedness in handling these challenging scenarios, ultimately improving patient care and outcomes.

One limitation of using virtual patient cases in medical education is the potential lack of complexity and variability that clinicians may encounter in real-world clinical settings. While virtual cases can simulate various scenarios and provide valuable learning experiences, they may not fully replicate the intricacies and unpredictability of actual patient encounters. Additionally, virtual patient cases may not encompass the cultural and contextual factors that can influence patient care and management decisions, especially in conditions like pelvic fistulas. Our hope is that this exposure will serve to stimulate interest in the topic of pelvic floor disorders, potentially motivating students to seek opportunities to gain hands-on exposure within and outside their required clerkships.

We identified several lessons learned when reflecting upon the design and implementation of our virtual cases. We found that providing the virtual cases in students’ rotation orientation email was an effective way to share them proactively. Sharing the cases again midway through the clerkship might improve student completion rates. Regardless of medical student year, most students valued the opportunity to engage with the cases as a means of preparing for real-life patient interactions and found the format conducive to achieving their learning objectives. Moreover, these cases can be shared with medical students of any level.

From a design perspective, we acknowledge that the cases require reliable internet access, which could be a barrier for some individuals. Incorporating free-text boxes for open-ended and reflective questions within the cases is an adaptation to consider in future case design to create a more user-friendly interface for students. We did not collect data on time required to complete the virtual cases or from faculty debrief sessions, which could have been insightful for future case design and planning.

Collecting feedback on the cases was challenging and is reflected in our overall low survey response rate. We recognize that our participant group included a small number of students across varying education years and that their heterogeneity limits the applicability of the results. Our survey was disseminated by email after students had completed their rotation and without a reminder. We did this to minimize the perception that responses could impact grades, but waiting until students had moved on to another rotation may have contributed to our low response rate. To collect more widespread data, we could disseminate the feedback survey prior to completion of the rotation and closer to the time students complete the cases.

The use of virtual and interactive urogynecologic patient cases on an e-learning platform represents an innovative approach to increasing clinical exposure to pelvic floor disorders. By providing medical students with the opportunity to interact with virtual patients, these cases can help bridge a gap in clinical education and prepare students to better diagnose and manage pelvic floor disorders in the future. Furthermore, open availability of the cases to students and educators is an essential first step in mitigating educational inequities, allowing for advancement in medical training and improvement in patient care.

## Appendices


Mrs. Smith - Rectovaginal Fistula folderMrs. Lopez - Vesicovaginal or Ureterovaginal Fistula folderGuide for Virtual Patient Cases.docxFeedback Survey.docx
*All appendices are peer reviewed as integral parts of the Original Publication.*
