# Supplementary material for: Virtual Education in Urogynecology: Enhancing Understanding and Management of Pelvic Fistulas
Source: MedEdPORTAL. 2024 Jun 4;20:11407. doi: 10.15766/mep_2374-8265.11407 (PMC11219081; doi:10.15766/mep_2374-8265.11407)
Supplement: Supplementary file 1 — Mrs. Smith - Rectovaginal Fistula folderMrs. Lopez - Vesicovaginal or Ureterovaginal Fistula folderGuide for Virtual Patient Cases.docxFeedback Survey.docx [file mep_2374-8265.11407-s001.zip › A. Mrs. Smith - Rectovaginal Fistula/content/assets/TWVCXY/Case A - Visit Summary.pdf]

Urogyn Case: Mrs. Smith
Visit Summary

Chief Complaint

Persistent brown vaginal discharge

HPI

Mrs. Smith is a 34 year-old woman who presents to the urogynecology clinic for an 8 month history of persistent brown vaginal discharge.

Characteristics of vaginal discharge

The vaginal discharge is persistent and a brown color, similar to the appearance of loose stool. It has a foul smell to it, and she is worried it could be stool. Sometimes, she will notice small particulate material in this brown vaginal discharge.

Duration

She first noticed the onset of the foul smelling, brown vaginal discharge a few days after a vaginal delivery 8 months prior, and it has been persistent since then. She notices the discharge throughout the day, every time she uses the toilet. She never had symptoms like this prior to her vaginal delivery.

Worsening/ Exacerbating factors

The brown vaginal discharge is worse when she has diarrhea or loose stools.

Alleviating factors

She cannot identify any significant alleviating factors.

Other associated symptoms

She does have issues controlling gas at times but unclear if this is coming from the anus or from the vagina.

Pad use/ Amount of discharge

She wears thin pads all day and changes them about 4x/day due to the brown discharge.

History of fecal incontinence

No history of fecal incontinence of solid or liquid stool. No diarrhea or constipation.

Number of bowel movements

She's had no issues or changes with her bowel movements. Typically has 1 bowel movement per day. With the aid of a Bristol stool chart during the interview, she points to "Type 4" as the main consistency of her typical bowel movements.

Obstetric history specific to this last vaginal delivery

She has a history of a 3rd degree laceration with her last delivery.

New medications since symptoms started

Denies any new medications.

Impact on quality of life

These symptoms have had a significant impact on her life. She is constantly worried about her discharge and is self-conscious that she smells like stool. She has stopped seeing friends and does not go to the gym anymore.

Impact on intercourse

She avoids intercourse because she is self-conscious about the discharge.

Prior evaluations or treatment

She has never taken medications for her symptoms or had prior evaluation with other health care providers for this specific issue.

Diet/ Fluid intake

Regarding her diet, she drinks 1 cup of regular iced-coffee in the morning, and 4 cups of water throughout the day.

Voiding issues

She has no issues with voiding. She voids approximately every 4 hours.

Other urinary tract symptoms

She denies any bladder symptoms.

Vaginal prolapse symptoms

No

Issues with vaginal or perineal splinting to complete defecation

No

Breastfeeding and childbearing status

The patient is no longer breastfeeding, and her menstrual cycles have returned. She has completed childbearing and her husband underwent a vasectomy.

Other Pertinent Questions/ History

Obstetric history

Miscarriage Ectopic pregnancy/ Abnormal pregnancy

1 spontaneous abortion (miscarriage at 6 weeks)

Deliveries

2014: Spontaneous abortion at 6 weeks of gestation.

2015: Spontaneous vaginal delivery (SVD) at 40 weeks of gestation. Male infant, 2600 grams. Delivery complicated by a 2nd degree perineal laceration. No episiotomy.

2017: SVD at 39 weeks. Female infant, 3700 grams, Delivery complicated by 2nd degree perineal laceration. No episiotomy.

8 months prior: SVD at 39 weeks. Male infant, 4100 grams. Delivery complicated by 3rd degree perineal laceration. No episiotomy.

Vaginal tear/ Episiotomy
History of a 3rd degree perineal laceration with her last vaginal delivery

Any other obstetric issues
No

Gynecologic history

Menarche
13 yo

Contraception
Husband underwent vasectomy 6 months prior

Menstrual history
Regular menses, q28 days

Last menstrual period (LMP)
10 days prior

History of pelvic infections/ Sexually transmitted infection (STI)/ Pelvic inflammatory disease (PID)
No history of infection.

Sexually active/ intercourse
Was sexually active until 3 months ago. She stopped having sex because she's self-conscious about the vaginal discharge. She has been in a monogamous relationship with her husband.

Dyspareunia
No issues or pain with sex.

Childbearing status
Has completed childbearing.

Last Pap smear
2 years ago and it was normal.

History of abnormal Pap smears

No

Any other GYN issues

No

Past medical history

Migraines without aura

Past surgical history

Wisdom teeth extraction

Medications

Multivitamin

Medication allergies

No allergies to medication.

Family history

None

Social history

Social history

Married, stay at home mom.

Drink alcohol

1-2 glasses of wine/week.

Smoke/ Tobacco history

Never smoked. No vaping.

Use any other recreational drugs

None.

Review of systems

A review of Mrs. Smith's systems shows that all other pertinent systems are negative except as mentioned previously.

Physical Examination

Vital signs

Heart rate

64

Respiratory rate

15

Blood pressure

117/66

Temperature

98.5 F

Pain score

0/10

Additional vital signs

Height

5 feet 4 inches

Weight

155 lbs

Body mass index (BMI)

25.7 kg/m^2

Physical examination parameters

General

Alert and oriented. No apparent distress (NAD).

Head and Neck

Normocephalic, Atraumatic

Cardiovascular
Regular rate and rhythm (RRR); no rubs, murmurs, or gallops

Pulmonary
Clear to auscultation bilaterally (CTAB); no wheezes, rhonchi, or rales

Abdomen
Soft, non tender, non distended. No guarding or rebound. No hepatosplenomegaly. No abdominal scars.

Lower extremities
Warm, well perfused bilateral lower extremities. No edema bilaterally. Palpable peripheral pulses bilaterally.

Rectal
Normal resting tone. Normal active tone. No masses noted. Scarring palpable in the anus/ rectum surrounding a 1 cm defect between vagina and anus/ rectum, just cephalad to the anal sphincter.

Pelvic
Parts of pelvic exam
Speculum exam
Bimanual exam

Pelvic exam
Normal appearing external female genitalia. Normal hair distribution. No clitoral enlargement. No skin changes, rashes, or lesions visualized.

Speculum exam
Normal appearing vagina and cervix with no masses or lesions. Brown, foul smelling vaginal discharge in the distal portion of vagina. Scarring noted on distal aspect of the posterior vaginal wall with a small, circular defect visualized (measuring 1 cm). This same defect was noted on rectal examination. There are no surrounding induration, inflammation, or evidence of purulent discharge.

Bimanual exam
Approximately 8 week size uterus, anteverted. No adnexal masses palpable.

Urogynecologic

Prolapse exam

No vaginal wall laxity on speculum exam.

Pelvic floor muscle strength

4/5

Fistula Suspicion

Clinic Procedure to be Performed: "Dye Gel Test"
Apply a few drops of blue dye to lubricating gel and gently apply the gel into the rectum with a finger. With a 1/2 speculum or retractor retracting the anterior wall of the vagina, the entire posterior vagina can be assessed for any evidence of blue-dyed gel.

In this particular patient, the fistula is easily visible. Thus, the dye gel test does not need to be performed.

You would perform this test if you suspected a fistula, but could not easily visualize it.

Other Physical Examination Findings/ Office Tests

Urine dip (Cost: $3 USD)/ Urinalysis (Cost: $45 - $247 USD)

Not indicated at this time.

***Note: Cost depends on insurance, location of lab, geography.**

Complete blood count (CBC) (Cost: $10 - $200 USD)

Not indicated at this time.

***Note: Cost depends on insurance, location of lab, geography.**

Basic metabolic profile (BMP) (Cost: $10 - $65 USD)

Not indicated at this time

***Note: Cost depends on insurance, location of lab, geography.**

Imaging Studies

Pelvic ultrasound (Cost: $195 - $700 USD)

Not indicated at this time.

***Note: Cost depends on insurance, location of lab, geography.**

Barium enema (Cost: $147 - $625 USD)

Not indicated at this time. If a fistula is not seen on physical examination with a suspicious history or multiple or complex fistula are expected, this test can be used to visualize defect(s).

***Note: Cost depends on insurance, location of lab, geography.**

Pelvic MRI (Cost: $1,000 - $5,000 USD)

Not indicated at this time. If a fistula is seen on physical examination with a suspicious history or multiple or complex fistula are expected, this test can be used to visualize defect(s). This test can also be used to visualize the internal and external anal sphincter if there is concern that the sphincter(s) may be disrupted.

***Note: Cost depends on insurance, location of lab, geography.**

Anal manometry (Cost: $800 - $1,283 USD)

Not indicated at this time.

***Note: Cost depends on insurance, location of lab, geography.**

Endoanal ultrasound (Cost: $57 - $112 USD)

Not indicated at this time. It can be considered if there is concern that the internal or external anal sphincters are disrupted.

***Note: Cost depends on insurance, location of lab, geography.**

Differential Diagnosis

- Fistula: Rectovaginal fistula
- Fistula: Fistula-in-ano
- Perineal abscess

- Vaginitis
- Fecal incontinence

Likely diagnosis

Rectovaginal fistula

Laboratory evaluation you could consider if no obvious fistula on exam, but evidence of abnormal discharge

It would be appropriate to workup abnormal vaginal discharge with a sexually transmitted infection (STI) panel.

Risk factor for developing symptoms

- Obstetric history with a history of 3rd degree perineal laceration

Non-surgical treatment options for symptoms

- Pelvic floor physical therapy
- Medications for stool bulking/firming such as fiber with fluid restriction
- Medications that slow bowel motility (e.g., loperamide, diphenoxylate, and atropine)

Surgical procedures to be considered for symptoms

- **Vaginal repair:** Rectovaginal fistula repair
- **Transanal repair:** Rectovaginal fistula repair with rectal mucosal advancement flap
- **Martius fibrofatty flap**
- **Anal sphincter repair**
