# Supplementary material for: Virtual Education in Urogynecology: Enhancing Understanding and Management of Pelvic Fistulas
Source: MedEdPORTAL. 2024 Jun 4;20:11407. doi: 10.15766/mep_2374-8265.11407 (PMC11219081; doi:10.15766/mep_2374-8265.11407)
Supplement: Supplementary file 1 — Mrs. Smith - Rectovaginal Fistula folderMrs. Lopez - Vesicovaginal or Ureterovaginal Fistula folderGuide for Virtual Patient Cases.docxFeedback Survey.docx [file mep_2374-8265.11407-s001.zip › B. Mrs. Lopez - Vesicovaginal or Ureterovaginal Fistula/content/assets/YD6Ooa/Case B - Visit Summary.pdf]

Urogyn Case: Mrs. Lopez
Visit Summary

Chief Complaint
Constant wetness

HPI
Mrs. Lopez is a 48 year-old woman who presents to the urogynecology clinic 3 weeks after a robotic hysterectomy reporting a constant feeling of wetness, soaking through her pants.

Characteristics of wetness
She reports it is yellow and has the odor of urine. The wetness is constant, throughout the day and also after she goes to sleep.

Duration of symptoms
This has been occurring for the past 6 or 7 days and started about 2 weeks after undergoing a hysterectomy.

Worsening/ exacerbating factors
None noticed.

Alleviating factors
None noticed.

Associated symptoms
She notes that there are air bubbles and blood in her urinary stream.

Pad use
Uses several pads during the daytime and a diaper at nighttime for sleep.

Other associated history

She reports undergoing a robotic-assisted laparoscopic hysterectomy for symptomatic fibroids about 3 weeks ago. She was told after surgery that there was "a lot of bleeding" and her "bladder was stuck".

Upon review of the operative note from outside hospital, the estimated blood loss was 500 mL and there were dense adhesions between the bladder and the uterus which were separated using electrocautery and blunt dissection. Cystoscopy was performed which revealed normal intact bladder and flow was seen from bilateral ureteral orifices.

Prior evaluations or treatments

Her primary gynecologist examined her the day after these symptoms began (postoperative day 14) and told her that she may have a problem with her bladder, but she would require specialty care to help her with this issue. The patient then presented to this clinic for further evaluation.

Other Pertinent Questions/ History

Obstetric history

Miscarriage Ectopic pregnancy/ Abnormal pregnancy

1998: Spontaneous abortion at 6 wks gestational age
2000: Missed abortion at 9 wks gestational age, required suction D&C
2003: Spontaneous abortion at 6 wks gestational age

Deliveries

2001: Spontaneous vaginal delivery (SVD) at 38 weeks of gestation, female, 3400g, Delivery complicated by a 2nd degree perineal laceration; No episiotomy
2004: Cesarean section with bilateral tubal ligation at 39 weeks of gestation for breech presentation, male 3500g

Vaginal tear/ Episiotomy

2001: 2nd degree perineal laceration; No episiotomy

How big was largest infant

3500g

Any other obstetric issues
No

Gynecologic history

Menarche
12yo

Contraception
Bilateral tubal ligation during cesarean section in 2004.

Menstrual history
Irregular, heavy bleeding related to uterine fibroids prior to hysterectomy.

Last menstrual period (LMP)
Unsure, they were very irregular.

History of pelvic infections/ Sexually transmitted infection (STI)/ Pelvic inflammatory disease (PID)
No history of infection.

Sexually active/ intercourse
Not currently due to postoperative restrictions.

Dyspareunia
No

Last Pap smear
Normal and obtained a month prior to hysterectomy.

History of abnormal Pap smears
No

Any other GYN issues
No

Past medical history

- Diabetes

- **Hypertension**
- **Obesity**

Past surgical history
- **Cesarean section – 2004**
- **Laparoscopic Cholecystectomy – 2014**
- **Robotic assisted total laparoscopic hysterectomy, bilateral salpingectomy – 3 weeks ago**

Medications
- **Metformin**
- **Lisinopril**

Medication allergies
Penicillin – the patient states she develops a rash.

Family history
None

Social history
Social history
Married, works at a manufacturing plant.

Drink alcohol
1-2 beers/week

Smoke/ Tobacco history
Smokes ½ pack/day.

Use any other recreational drugs
None

Review of systems
Positive for chronic lower back pain. All other pertinent review of systems is negative, except as mentioned previously.

Physical Examination

Vital signs

Heart rate
72 bpm

Respiratory rate
16 bpm

Blood pressure
138/76 mmHg

Temperature
98.7 F

Pain score
2/10

Additional vital signs

Height
5 feet 3 inches

Weight
186 lbs

Body mass index (BMI)
33 kg/m^2

Physical examination parameters

General
Mildly anxious and somewhat uncomfortable but not in acute distress.

Head and Neck
Normocephalic, Atraumatic

Cardiovascular
Regular rate and rhythm (RRR); no rubs, murmurs, or gallops

Pulmonary
Clear to auscultation bilaterally (CTAB); no wheezes, rhonchi, or rales

Abdomen
Multiple small 1-2 cm surgical incisions are clean, dry and intact, no erythema , edema or induration. Soft, obese, no distention, slight tenderness with palpation but no rebound/ guarding.

Lower extremities
Warm, well perfused bilateral lower extremity. No edema bilaterally. Palpable peripheral pulses bilaterally.

Rectal
No masses.

Pelvic
Parts of pelvic exam
Speculum exam
Bimanual exam

Pelvic exam
Normal appearing external female genitalia. Normal hair distribution. No clitoral enlargement. No skin changes, rashes, or lesions visualized. There is excess moisture noted.

Speculum exam
Normal appearing intact vaginal cuff. Pooling of clear yellow liquid in the apex of the vagina. There is no surrounding inflammation or evidence of purulent drainage.

Bimanual exam
Slightly tender, but intact, vaginal cuff with induration. No discrete mass palpable. No clear defect appreciable.

Urogynecologic
Prolapse exam
No vaginal wall laxity on speculum exam.

Pelvic floor muscle strength

1/5

Post void residual

10ml

Tampon test (Cost: $5 - $50 USD)

After the catheter is placed in the bladder and the bladder emptied to determine the post void residual, a tampon or gauze is placed in the vagina and then the bladder is retrograde filled with colored sterile water or saline (e.g., 200 ml of water or saline mixed with a few drops of methylene blue). The tampon/ gauze is then removed and it is noted to be wet and light blue in color.

***Note: Cost depends on insurance, location of lab, geography.**

Other Physical Examination Findings/ Office Tests

Urine dip (Cost: $3 USD)/ Urinalysis (Cost: $45 - $247 USD)

	Patient Value	Normal Values
Color	Yellow	Yellow (light/pale to dark/deep amber)
Clarity	Clear	Clear or cloudy
pH	7	4.5 – 8
Specific gravity	1.03	1.005 – 1.025
Glucose	+	$\leq$ 130 mg/d
Ketones	Negative	Negative
Nitrites	Negative	Negative
Leukocyte esterase	Moderate	Negative

**Blood**	**Large**	**Negative**
**Bilirubin**	**Negative**	**Negative**
**Urobilirubin**	**Negative**	**Small amount (0.5 – 1 mg/dL)**

***Note: Cost depends on insurance, location of lab, geography.**

Creatinine test of vaginal fluid (Cost: $25 - $74 USD)

Vaginal fluid is collected in a specimen cup and then sent for laboratory testing for the creatinine level.

***Note: Cost depends on insurance, location of lab, geography.**

Complete blood count (CBC) (Cost: $10 - $200 USD)

Not indicated at this time.

***Note: Cost depends on insurance, location of lab, geography.**

Basic metabolic profile (BMP) (Cost: $10 - $65 USD)

Not indicated at this time

***Note: Cost depends on insurance, location of lab, geography.**

Office Procedures

Cystourethroscopy(Cost: $350 - $3,000 USD)

You are able to perform this procedure on the same day as her clinic visit and found scarring in the posterior bladder with possible defect, measuring about 0.5 - 1 cm, supratrigonal and midline, about 2cm from either ureteral orifice. With a finger in the vagina, you can feel cystoscopic fluid draining from this area.

***Note: Cost depends on insurance, location of lab, geography.**

Urodynamic testing (Cost: $350 - $1,000 USD)

Not indicated at this time.

***Note: Cost depends on insurance, location of lab, geography.**

Imaging Studies

Pelvic ultrasound (Cost: $195 - $700 USD)

Not indicated at this time.

***Note: Cost depends on insurance, location of lab, geography.**

CT urogram (Cost: $1,700 - $10,000 USD)

This is indicated in this patient for evaluation of ureters even if there is a defect seen in the bladder on cystourethroscopy because it is estimated that 12% of women with defects in the bladder after hysterectomy also have ureteral compromise. Retrograde pyelograms and voiding cystograms can also be utilized if CT not feasible.

***Note: Cost depends on insurance, location of lab, geography.**

Pelvic MRI (Cost: $1,000 - $5,000 USD)

Not indicated at this time.

***Note: Cost depends on insurance, location of lab, geography.**

Differential Diagnosis

- Fistula: Vesicovaginal fistula
- Fistula: Ureterovaginal fistula
- Pelvic abscess
- Vaginal discharge
- Mixed Urinary incontinence
- Stress incontinence
- Urge incontinence
- Overflow incontinence
- Urinary tract infection

Likely diagnoses

- **Fistula: Vesicovaginal fistula**
- **Fistula: Ureterovaginal fistula**

Main risk factors for developing symptoms

- **Recent robotic-assisted total laparoscopic hysterectomy, bilateral salpingectomy**
- **Diabetes**
- **Smoking**

Other risk factors (that Mrs. Lopez does not have) for developing these types of fistulas

- **Prior history of a fistula**
- **Prior history of reconstructive or mesh surgery**
- **Radiation**
- **Trauma**
- **Cancer**

First line non-surgical treatment options for symptoms

- **Transurethral Foley catheter placement for continuous bladder drainage (usually for 3-4 weeks)**

Surgical procedures to be considered for symptoms

- **Cystoscopic repair: placement of sealant through the cystoscope and into the fistula tract usually following mechanical curettage of the fistula tract**
- **Vaginal repair: Latzko partial colpocleisis +/- Martius flap**
- **Transabdominal (laparotomy/laparoscopic/robotic) fistula repair +/- omental flap**
