# Supplementary material for: Virtual Education in Urogynecology: Enhancing Understanding and Management of Pelvic Fistulas
Source: MedEdPORTAL. 2024 Jun 4;20:11407. doi: 10.15766/mep_2374-8265.11407 (PMC11219081; doi:10.15766/mep_2374-8265.11407)
Supplement: Supplementary file 1 — Mrs. Smith - Rectovaginal Fistula folderMrs. Lopez - Vesicovaginal or Ureterovaginal Fistula folderGuide for Virtual Patient Cases.docxFeedback Survey.docx [file mep_2374-8265.11407-s001.zip › B. Mrs. Lopez - Vesicovaginal or Ureterovaginal Fistula/content/index.html]

Case B
