# Supplementary material for: Virtual Education in Urogynecology: Enhancing Understanding and Management of Pelvic Fistulas
Source: MedEdPORTAL. 2024 Jun 4;20:11407. doi: 10.15766/mep_2374-8265.11407 (PMC11219081; doi:10.15766/mep_2374-8265.11407)
Supplement: Supplementary file 1 — Mrs. Smith - Rectovaginal Fistula folderMrs. Lopez - Vesicovaginal or Ureterovaginal Fistula folderGuide for Virtual Patient Cases.docxFeedback Survey.docx [file mep_2374-8265.11407-s001.zip › C. Guide for Virtual Patient Cases.docx]

**Medical Student Virtual Urogynecology Patient Cases Guide**

**General instructions:**

To maximize your learning in reviewing the virtual patient cases, we recommend the following to be completed in the order listed:

1. Read the optional background materials (listed below) in advance of completing the online virtual patient cases.
   1. Approximate time allotment: 15 minutes per article.
2. Work through online modules (Appendix A: Mrs. Smith: rectovaginal fistula and Appendix B: Mrs. Lopez: vesicovaginal fistula or ureterovaginal fistula).
   1. Detailed instructions for accessing modules/cases listed below.
   2. Approximate time allotment: 30 minutes per case, variable depending on time allotted for discussion.
3. Review case summary document after completing online modules.
   1. Approximate time allotment: 3 minutes per case.
4. Arrange time with your gynecology/ urogynecology faculty or fellow to discuss and debrief on these cases (optional).

**Optional background readings/ materials**

1. Pelvic organ prolapse. ACOG Clinical. November 2019. Practice Bulletin 214.
   1. <https://www.acog.org/clinical/clinical-guidance/practice-bulletin/articles/2019/11/pelvic-organ-prolapse>
2. Urinary incontinence in women. ACOG Clinical. November 2015. Practice Bulletin 155.
   1. <https://www.acog.org/clinical/clinical-guidance/practice-bulletin/articles/2015/11/urinary-incontinence-in-women>
3. Evaluation of uncomplicated stress urinary incontinence in women before surgical treatment. ACOG Clinical. June 2014. Committee Opinion 603.
   1. <https://www.acog.org/clinical/clinical-guidance/committee-opinion/articles/2014/06/evaluation-of-uncomplicated-stress-urinary-incontinence-in-women-before-surgical-treatment>
4. Fecal incontinence. ACOG Clinical. April 2019. Practice Bulletin 210.
   1. <https://www.acog.org/clinical/clinical-guidance/practice-bulletin/articles/2019/04/fecal-incontinence>
5. *Fistulas: Genitourinary and Rectovaginal.* American Urogynecologic Society; 2023.
   1. <https://www.voicesforpfd.org/assets/2/6/Fistulas.pdf>

**Instructions for accessing and completing online modules/cases:**

1. Download the zip file Appendices (A and B) to access virtual modules.
2. Click the .html link within the downloaded zip file to access each virtual module webpage.
3. Cases were designed to be completed individually on the student’s own time but can be completed in a group with discussion as questions are embedded throughout the case.
4. Each module begins with a chief complaint with interactive prompts to navigate through the case.
5. The learner will be prompted to think of questions they would ask (i.e., What other pertinent health history do you need to complete before the physical examination?).
6. Reflective questions are also embedded within the modules (i.e., What questions did you not ask? What findings did you gather from questions you did not originally ask that you think will be relevant moving forward?).
7. We recommend navigating through the online case with a notebook to record their thoughts and questions.
8. After completing the online case, we recommend reviewing the summary document.
