# Supplementary material for: Virtual Education in Urogynecology: Enhancing Understanding and Management of Pelvic Fistulas
Source: MedEdPORTAL. 2024 Jun 4;20:11407. doi: 10.15766/mep_2374-8265.11407 (PMC11219081; doi:10.15766/mep_2374-8265.11407)
Supplement: Supplementary file 1 — Mrs. Smith - Rectovaginal Fistula folderMrs. Lopez - Vesicovaginal or Ureterovaginal Fistula folderGuide for Virtual Patient Cases.docxFeedback Survey.docx [file mep_2374-8265.11407-s001.zip › D. Feedback Survey.docx]

**Survey for Virtual Cases**

**Demographic Questions:**

1. What gender do you identify as?

- Female
- Male
- Nonbinary / third gender
- Prefer not to say

1. What is your age?

- _________

1. Have you had experience with any other exclusively online learning courses before completing the virtual cases?

- Yes, prior to COVID-19
- Yes, since COVID-19
- No
- Other (please specify): _______________

1. In which year of medical school did you complete the virtual patient cases?

- Pre-med
- 1st year
- 2nd year
- 3rd year
- 4th year
- Other (please specify): __________________

1. What medical specialty are you planning to pursue?

- Anesthesiology
- Dermatology
- Emergency Medicine
- Family Medicine
- Internal Medicine
- Neurology
- Ob/Gyn
- Pathology
- Pediatrics
- Psychiatry
- Radiation Oncology
- Radiology
- Surgical-related specialty (General Surgery, Orthopedics, Otolaryngology, Ophthalmology, Neurosurgery, Urology, Plastics, Vascular, etc).
- Undecided
- Other (please specify): _______________________

1. Which medical rotations have you completed (prior to completing the virtual cases)? (select all that apply)

- Pre-med only
- Pre-clinical work only
- Anesthesiology
- Dermatology
- Emergency Medicine
- Family Medicine
- Internal Medicine
- Neurology
- Ob/Gyn
- Pathology
- Pediatrics
- Psychiatry
- Radiation Oncology
- Radiology
- Surgical-related specialty (General Surgery, Orthopedics, Otolaryngology, Ophthalmology, Neurosurgery, Urology, Plastics, Vascular, etc).
- Other (please specify): _______

**Case-Related Questions:**

Please evaluate the following statements in regard to your experience with the virtual cases.

1. The format of the virtual cases was easy to use.


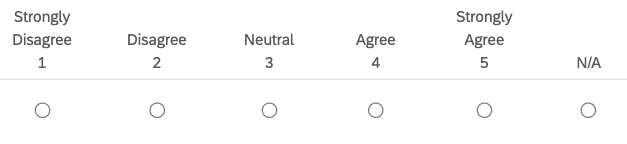


1. The format of the virtual cases was effective at accomplishing my learning goals.


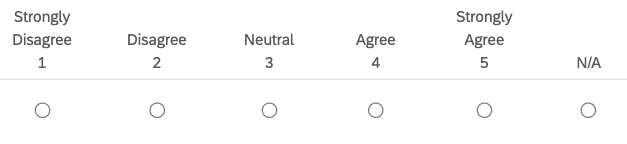


1. The virtual cases were appropriate for my level of learning.


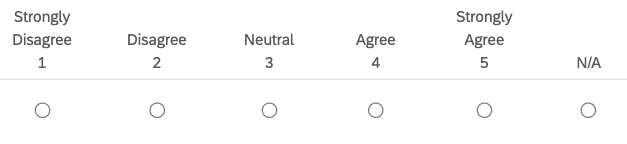


1. The additional case resources were useful in supplementing my learning.


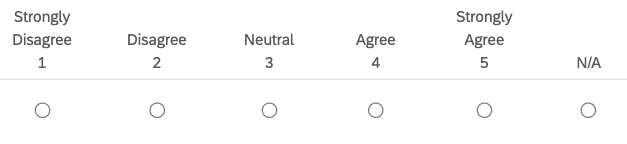


1. The knowledge/skills that I gained from the virtual cases will be useful in my future career.


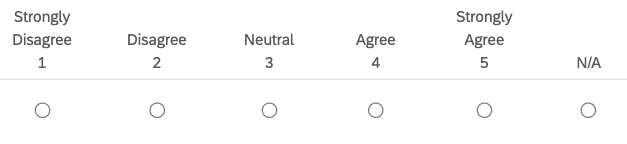


1. The virtual cases were useful in advancing my knowledge of urogynecology and pelvic floor disorders.


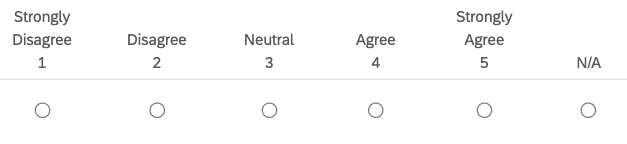


1. The virtual cases increased my interest in urogynecology and pelvic floor disorders.


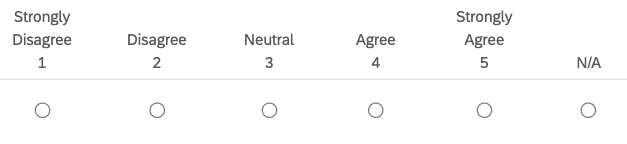


1. The virtual cases can take the place of an in-person urogynecology rotation.


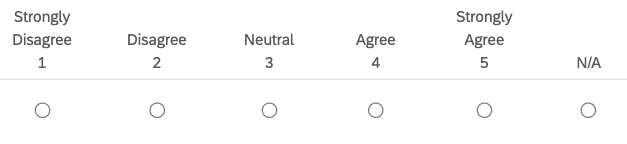


1. The virtual cases can shorten the duration of an in-person urogynecology rotation.


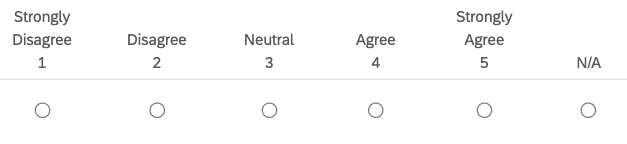


1. I would recommend the virtual cases to other students.


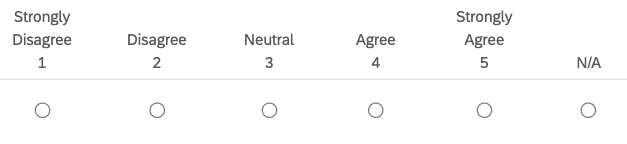


1. As an overall impression, I was satisfied with the virtual cases.


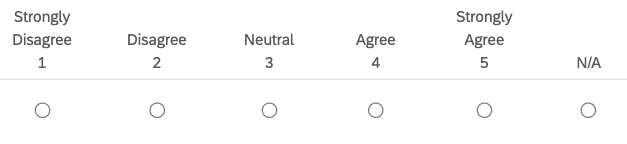


**Self-Assessment Questions:**

Compared with your own baseline comfort level with each of the following topics, how did completing the virtual cases affect your progress towards the following learning objectives?

1. Non-surgical management options for pelvic fistula


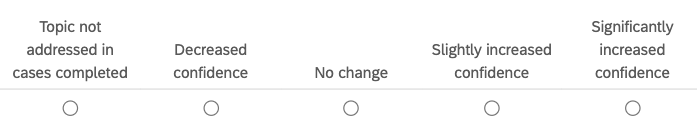


1. Surgical management options for pelvic fistula


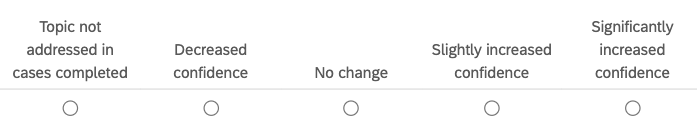


1. Identifying risk factors associated with development of a fistula


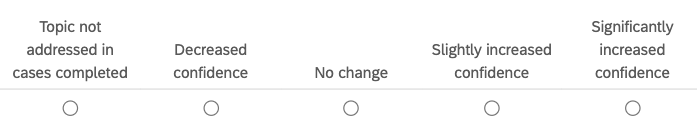


1. Awareness of cost associated with laboratory and imaging work-ups commonly performed in urogynecology


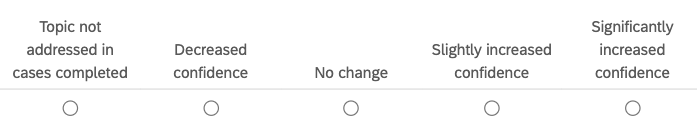


**Open-Ended Questions:**

1. What did you enjoy about the virtual cases?
2. What did you dislike about the virtual cases?
3. What did you find to be the pros and cons of the virtual case format vs. an in-person rotation? Please elaborate if applicable.
4. Do you have any suggestions for improving the virtual cases?
